# COVID-19 mitigation measures to maintain access to essential health services: new opportunities with long-term benefits

**DOI:** 10.11604/pamj.2021.40.254.31264

**Published:** 2021-12-21

**Authors:** Jacquelyn Lickness, Pamela Bachanas, Rania Tohme, Anna Russell, Allen Craig, Avi Hakim

**Affiliations:** 1 Centers for Disease Control and Prevention, Center for Global Health, Division of Global HIV and TB, 4 Le Duan Street, District 1, Ho Chi Minh City, Vietnam,; 2 Centers for Disease Control and Prevention, Center for Global Health, Division of Global HIV and TB, 3 Corporate Boulevard, Atlanta, GA 30320, USA,; 3 Centers for Disease Control and Prevention, National Center for Immunization and Respiratory Diseases, Division of Bacterial Diseases, 1600 Clifton Rd NE, Atlanta, GA 30329

**Keywords:** COVID-19, health services, task shifting, telehealth

## Abstract

The coronavirus disease 2019 (COVID-19) pandemic has substantially impacted health systems globally. To highlight potential opportunities to improve health service delivery in low- and middle-income countries, we describe lessons learned from published literature and experiences responding to the pandemic. The benefits of healthcare service measures implemented during the pandemic with potential for lasting benefits for strengthening health systems are highlighted: 1) innovative pharmaceutical dispensing methods; 2) appointment-based systems in health facilities; 3) telehealth to provide patient care; 4) task shifting to redistribute healthcare workloads; and 5) home-based pulse oximetry to monitor oxygen levels. These measures can reduce unnecessary contact with healthcare staff while maintaining critical health service delivery and may be of value to continue after the pandemic subsides.

## Commentary

The coronavirus disease 2019 (COVID-19) pandemic has substantially impacted healthcare systems globally. Healthcare resources and staff are diverted to screen, test, and care for persons with COVID-19, and there are numerous reports of personal protective equipment (PPE) shortages. Low resource settings are particularly vulnerable to the impacts of COVID-19 due to resource constraints, high burden of other infectious diseases, and weakness in the healthcare infrastructure. Hospital visits and care for non-COVID-19-related medical issues decreased in many countries during the pandemic, both due to movement restrictions as a result of lockdown policies and because patients avoided health facilities for fear of being infected with severe acute respiratory syndrome coronavirus 2 (SARS-CoV-2), the virus that causes COVID-19 [1]. In addition, many healthcare personnel (HCP) were infected with SARS-CoV-2 or quarantined due to exposures. Recent models estimated that in countries with high burdens of HIV infection, tuberculosis (TB), and malaria, deaths from these diseases over 5 years may increase by up to 10%, 20%, and 36%, respectively, compared to pre-COVID-19 years due to interruptions in service delivery [2].

Despite the negative impact of COVID-19 on global healthcare systems, the pandemic presents opportunities to modify service delivery measures for people with chronic illnesses or mild to moderate COVID-19 symptoms. We reviewed literature published about Africa and Southeast Asia between January 2020 and July 2021 that presented information on health system responses to COVID-19. Additionally, we captured information from public health experts from the U.S. Centers for Disease Control and Prevention (CDC) and partner organizations working in low- and middle-income countries (LMIC). We highlight the benefits of five major healthcare service modifications implemented in response to COVID-19 that could also be scaled up for long-term use among people with chronic illnesses or mild to moderate COVID-19 symptoms: 1) dispensing multiple months of medications to patients to decrease pharmacy and clinic visits and fast tracking treatment services; 2) introducing appointment-based systems in health facilities to ensure social distancing in waiting rooms; 3) adopting or expanding the use of telehealth to provide patient care; 4) implementing task shifting to redistribute workloads of HCP; and 5) using pulse oximetry in non-clinical settings. All five interventions help mitigate the risk of COVID-19 transmission and other communicable diseases in healthcare settings while ensuring the continuity of service delivery [3,4].

**Innovate medication delivery:** innovative medication delivery includes alternative models such as multi-month dispensing, “fast track” pharmacies, and community pick-up points. Such models are particularly useful for persons requiring frequent follow-up with the healthcare system, including elderly persons or those with chronic illnesses. Exposure to COVID-19 patients can be reduced in typical healthcare facilities in low- and middle-income countries (LMICs) by adapting service delivery models. HIV programs in sub-Saharan Africa began providing spaced clinic visits and multi-month medication dispensing for patients in health facilities and community locations such as markets, churches, and local pharmacies before the COVID-19 pandemic [5].

For patients who need or prefer to come to the healthcare facility to pick up medications, “fast-track” services reduce time spent in the facility and contact with HCP and other patients. Such services include a fast-track lane at the pharmacy where medical charts are pulled in advance of the visit, refills are pre-prepared, patients arrive at a specific time to pick up the medications and are screened for symptoms but do not see the HCP if well. Fast-track pharmaceutical services have been implemented in multiple countries; in Malawi, this model demonstrated greater retention and adherence to treatment [6]. This approach could be continued after the pandemic to reduce patient and HCP exposure risk to other communicable diseases (e.g., TB, influenza), decongest clinics, give HCP more time to focus on sicker patients, and reduce patients´ cost, transportation time to the clinic, and missed time from work and school.

**Implement appointment times:** many health facilities in low resource settings offer services during a pre-determined block of time, typically during morning hours, on a first come, first served basis. This often results in overcrowded waiting rooms and long wait times, increasing the risk of exposure to infectious diseases. Implementing and enforcing scheduling systems with specific appointment days and times decongests crowded waiting areas and allows physical spacing to reduce potential exposures. A study in Mozambique evaluated the effectiveness of appointment scheduling in two urban and one rural high-volume antenatal clinics. The study demonstrated that using appointment scheduling could reduce patient wait time by up to 100 minutes [7]. This approach can be used after the pandemic to decrease waiting times, improve patient experience with the healthcare system, and reduce risk of exposure to infectious diseases.

**Implement and scale-up telehealth:** telehealth is the use of electronic information and telecommunications technologies to support and promote long-distance clinical healthcare, patient and professional health-related education, and public health. Telehealth mitigates the spread of disease, preserves valuable PPE and maintains healthcare worker and patient safety while addressing patient needs [8]. Telehealth substantially increased during COVID-19, especially in countries with pre-existing telehealth platforms and regulations. Telehealth was effectively implemented to minimize disruption of noncommunicable disease care in almost 60% of the 120 countries reporting challenges in service delivery [9]. In sub-Saharan Africa, where over 80% of people have access to mobile phones, telehealth technology is being used during the pandemic to triage patients before they enter the hospital, conduct contact tracing and screen contacts, remotely monitor patients, and educate the public and HCP. Some countries have initiated/piloted novel systems (Vietnam), expanded pre-existing platforms from other diseases for health promotion (Mozambique), or utilized low-tech phones for contact tracing using existing software from Ebola outbreaks (Liberia) [10]. Telehealth will likely be used after the pandemic for both emergencies and routine patient care to save time and money, and provide more efficient healthcare services.

**Implement task shifting:** low-resource settings often face shortages in qualified healthcare workers. Task shifting is the redistribution of tasks to HCP with less training and fewer qualifications, potentially freeing time of more highly skilled HCP to provide quality care. For example, community-based health workers can be trained in a new skill or area of work, which in turn allows routine health services to be more easily accessed by patients within their community. Task shifting has the potential to improve integration of health services by promoting a greater exchange and cross-training across specialties and has demonstrated an increased efficiency and cost savings [3]. Countries have implemented task shifting in health settings for decades in response to human resource shortages during outbreaks or as a mechanism to deliver routine health services in chronically understaffed health facilities. Countries implemented task shifting during the COVID-19 pandemic to ensure sufficient staff were available to attend to patients. Some countries have reassigned or relocated healthcare workers from their regular duties (Zimbabwe, Nigeria), while others have expanded the scope of community health workers to assist with COVID-19 activities like contact tracing or risk communication (Rwanda, Ethiopia) (Pam Bachanas 2020, personal communication, June). Introducing or expanding the use of task shifting has the potential to yield long-term advantages after the pandemic by increasing the number of skilled and cross-trained health workers while also broadening access to care, especially among those most difficult to reach.

**Use of pulse oximetry:** pulse oximetry is a non-invasive test that measures the oxygen level in a patient´s blood. A phenomenon called “silent hypoxemia”, whereby patients have low blood oxygen levels without shortness of breath, has emerged as an important risk for persons with COVID-19. Approximately 17% of persons infected with SARS-CoV-2 will require clinical care, which often includes supplemental oxygen and for severe cases, mechanical ventilation [1]. Monitoring oxygen saturation through pulse oximetry is critical, low-cost method for the early recognition and treatment of hypoxia in these persons. Home use of pulse oximetry has helped to reduce healthcare utilization and mortality because patients can monitor their oxygen levels and communicate with their physician in the event of any changes [4]. In low resource settings where healthcare resources like hospital beds and supplemental oxygen are limited, widespread adoption of pulse oximetry during the COVID-19 pandemic could alleviate patient volume in healthcare settings. In communities that have pulse oximeters with people trained to use them, patients and caregivers can identify who should be referred to a higher level of care or who are safe to remain in isolation at home, thereby reducing unnecessary use of advanced care resources. Pulse oximetry is a potential tool to manage other respiratory illnesses in non-clinical settings. Its successful use can also lay a foundation for the use of other simple tools by community health workers during the pandemic and after.

**Challenges in implementation:** the measures proposed here are subject to several limitations. First, changes in service delivery and introduction of interventions may require review and possible modifications or development of national guidelines and policies. For example, use of telehealth might require revisions to the country´s healthcare services and reimbursement policies. Second, the need for additional human, financial, or technical resources may be a barrier to implementation or may take considerable time to scale up in certain settings. For example, task shifting requires developing and rolling out training and continuous monitoring and both home-based pulse oximetry and telehealth require investment in technological infrastructure. Third, there may be limitations in the application of some interventions for both healthcare facilities and patients. For example, telehealth cannot replace all health services as in-person visits may be necessary for some illnesses. Some patients may have difficulties accessing telehealth platforms or web-based scheduling systems. Additionally, implementing time-based appointment systems may be difficult, as patients´ availability may be limited because of work or other duties.

## Conclusion

The COVID-19 pandemic has disrupted healthcare systems, affecting access to care, and creating strains on HCP and service delivery. The measures highlighted in this paper can assist in mitigating the transmission of SARS-CoV-2 while positively impacting health systems beyond the pandemic. These adaptations to the health systems implemented during the COVID-19 pandemic are considerations for long-term strategies to improve the effectiveness and efficiency of health service delivery in low- and middle-income settings.
